# *Atlantia*, a new genus of Dendrophylliidae (Cnidaria, Anthozoa, Scleractinia) from the eastern Atlantic

**DOI:** 10.7717/peerj.8633

**Published:** 2020-03-16

**Authors:** Kátia C.C. Capel, Cataixa López, Irene Moltó-Martín, Carla Zilberberg, Joel C. Creed, Ingrid S.S. Knapp, Mariano Hernández, Zac H. Forsman, Robert J. Toonen, Marcelo V. Kitahara

**Affiliations:** 1 Centro de Biologia Marinha, Universidade de São Paulo, São Sebastião, São Paulo, Brazil; 2 Coral-Sol Research, Technological Development and Innovation Network, Rio de Janeiro, Brazil; 3 Departamento de Biología Animal, Edafología y Geología. Facultad de Ciencias, Universidad de La Laguna, San Cristóbal de La Laguna, Canary Islands, Spain; 4 Departamento de Bioquímica, Microbiología, Biología Celular y Genética, Facultad de Ciencias, Instituto Universitario de Enfermedades Tropicales y Salud Pública de Canarias, Universidad de La Laguna, San Cristóbal de La Laguna, Canary Islands, Spain; 5 Instituto de Biodiversidade e Sustentabilidade, Universidade Federal do Rio de Janeiro, Macaé, Rio de Janeiro, Brazil; 6 Departamento de Ecologia, Universidade do Estado do Rio de Janeiro, Rio de Janeiro, Brazil; 7 School of Ocean & Earth Science & Technology, Hawai’i Institute of Marine Biology, University of Hawai’i at Mānoa, Kaneohe, Hawai’i, United States of America; 8 Departamento de Ciências do Mar, Universidade Federal de São Paulo, Santos, São Paulo, Brazil

**Keywords:** Azooxanthellate corals, Tubastraea, Cape verde

## Abstract

*Atlantia* is described as a new genus pertaining to the family Dendrophylliidae (Anthozoa, Scleractinia) based on specimens from Cape Verde, eastern Atlantic. This taxon was first recognized as *Enallopsammia micranthus* and later described as a new species, *Tubastraea caboverdiana*, which then changed the status of the genus *Tubastraea* as native to the Atlantic Ocean. Here, based on morphological and molecular analyses, we compare fresh material of *T. caboverdiana* to other dendrophylliid genera and describe it as a new genus named *Atlantia* in order to better accommodate this species. Evolutionary reconstruction based on two mitochondrial and one nuclear marker for 67 dendrophylliids and one poritid species recovered *A. caboverdiana* as an isolated clade not related to *Tubastraea* and more closely related to *Dendrophyllia cornigera* and *Leptopsammia pruvoti*. *Atlantia* differs from *Tubastraea* by having a phaceloid to dendroid growth form with new corallites budding at an acute angle from the theca of a parent corallite. The genus also has normally arranged septa (not Portualès Plan), poorly developed columella, and a shallow-water distribution all supporting the classification as a new genus. Our results corroborate the monophyly of the genus *Tubastraea* and reiterate the Atlantic non-indigenous status for the genus. In the light of the results presented herein, we recommend an extensive review of shallow-water dendrophylliids from the Eastern Atlantic.

## Introduction

Comprising 22 extant genera and 171 extant species, the family Dendrophylliidae Gray, 1847 is the third most speciose of the order Scleractinia (Hoeksema & Cairns, 2018). Such diversity is represented by a wide variety of growth forms (e.g., solitary and colonial), presence or absence of algal symbionts (i.e., zooxanthellate, azooxanthellate and apozooxanthellate), and an extensive geographic and bathymetric ranges, occurring from the tropics to polar regions at depths up to 2,165 m (Cairns, 2001). Although the family was recovered as monophyletic in the light of molecular data (Kitahara et al., 2010; Arrigoni et al., 2014), the generic evolutionary relationships within the family remains unclear, including several poly/paraphyletic genera (Arrigoni et al., 2014; Kitahara et al., 2016).

The classical taxonomy of scleractinian corals relies on skeletal morphological characters, but high intraspecific variation, convergence and homoplasy frequently challenge their identification, especially in shallow-water species (Todd, 2008). Among dendrophylliids, morphological characters used to reconstruct the evolutionary history of the group (i.e., corallum morphology, theca structure, calicular elements, and presence of zooxanthellae) do not seem to be sufficiently informative (Arrigoni et al., 2014). In addition, not all evolutionary changes resulting in speciation, such as changes in reproduction and ecology, are accompanied by detected morphological changes (Paz-García, García-de León & Balart, 2015; Gélin et al., 2017).

The genus *Tubastraea* Lesson, 1829 currently comprises seven extant species and several unidentified morphotypes, all azooxanthellate, six of which are native to the Eastern Pacific or Indo-Pacific Oceans (Cairns, 2001; Fenner, 2005; Arrigoni et al., 2014), and one recently described as endemic to Cape Verde, in the eastern Atlantic (EA) (Ocaña et al., 2015). However, the taxonomic status of *Tubastraea* in the EA is unclear and has been discussed for more than four decades (Laborel, 1974; Creed et al., 2017). Laborel (1974) examined the distribution and taxonomy of the shallow-water corals from EA and recorded the occurrence of *Tubastraea* from the Gulf of Guinea, Gabon, Sierra Leone and Cape Verde, suggesting that the genus was recently introduced from the Indo-Pacific or the Caribbean. Fossils of *T. coccinea* Lesson, 1830 have been reported from Pleistocene substrates of Cape Verde (Boekschoten & Best, 1988). However, no description or figures were provided to support this claim. At Gulf of Guinea, Gabon, and Sierra Leone two distinct morphotypes differing by colony growth form and tissue pigmentation were mentioned by Laborel (1974), one bearing orange subplocoid colonies and the other displaying a branching colony and a yellow coenosarc. Nevertheless, apart from displaying either orange or yellow tissue pigmentation, two branching morphs indistinguishable by traditional skeleton characters were observed at Cape Verde, resembling the yellow form found at the Gulf of Guinea (Laborel, 1974).

Historically, specimens from Cape Verde were first identified as *Enallopsammia micranthus* (Ehrenberg, 1834) by Chevalier (1966) due to their dendroid colony growth form; however, in a revision of this genus Zibrowius (1973) considered it a different species, more closely related to *Coenopsammia* Milne, Edwards & Haime, 1848 but differing from the Indo-Pacific morphs. The genus *Coenopsammia* was later synonymized to *Tubastraea* (Cairns, 2001). Laborel (1974) recognized the Cape Verde species as *Tubastraea* sp., but highlighted the need for a taxonomic revision of the whole genus. More recently, Ocaña et al. (2015) re-examined specimens from Cape Verde and described a new species, *Tubastrea caboverdiana* Ocaña & Brito, 2015 (with a wrong spelling of the genus name), based on morphological differences from other *Tubastraea* species, especially *T. coccinea*. However, the authors highlighted the need for re-evaluation of the EA shallow-water dendrophylliids. Recently, new occurrences of introduced *T. coccinea* and *T. tagusensis* were recorded in the Canary Islands, EA, where it seems to be spreading quickly from artificial to natural substrates (Brito et al., 2017; López et al., 2019).

Due to the controversial status of the genus *Tubastraea* in the Atlantic Ocean, specimens of *T. caboverdiana* were sampled for molecular analyses, additionally to morphological comparison, in order to confirm their species identity. Following the examination of new samples from the type locality we observed several morphological characters inconsistent with those of *Tubastraea*. Such morphological divergence is mirrored at the molecular level, and together indicate that *T. caboverdiana* represents an undescribed dendrophylliid genus. Here we describe the new genus and discuss the main morphological divergences in relation to other genera of the same family.

## Material & Methods

### Sampling

A total of 21 specimens from both color morphotypes (ten orange and eleven yellow colonies) were collected by SCUBA diving at 6 to 10 m depth at Tarrafal, Santiago Island, Cape Verde—15°10′N, 23°47′W (type locality of *T. caboverdiana*) in April 2015 and four additional specimens were collected from both natural and artificial substrates at four sites of Mindelo, São Vicente Island at 1 to 14 m depth in November 2017 (the study was carried out under authorization No. 014/2015 from the Direcção Nacional do Ambiente, Cabo Verde). Tissue samples from each colony were preserved in CHAOS solution (4 M guanidine thiocyanate, 0.1% N-lauroyl sarcosine sodium, 10 mM Tris pH 8, 0.1 M 2-mercaptoethanol) (Fukami et al., 2004) or absolute ethanol for molecular analyses, and the skeleton of specimens collected at Santiago Island were bleached in a sodium hypochlorite solution for morphological analyses. All dry specimens from Santiago Island are deposited at the Museu Nacional do Rio de Janeiro (MNRJ 9108-9111; 9113-9115) (File S1).

### Morphological comparison

The species re-description was based on the newly sampled specimens and also on pictures of the holotype of *T. caboverdiana*, deposited at the *Museo del mar de Ceuta* (MMC) (Spain) (MMC-26) (Ocaña et al., 2015). Identification and comparison to other Dendrophylliidae followed Chevalier (1966), Zibrowius (1980), Cairns (2001), and Cairns & Kitahara (2012). One small polyp from each color morph was separated for Scanning Electron Microscopy (SEM) images. Polyps were fixed on stubs using double side adhesive tapes, subjected to gold coating, and visualized under the microscopy JEOL, model JSM-6510 from the Laboratory of Images in Optical and Electronic Microscopy of the Institute of Biology at the Federal University of Rio de Janeiro.

### Nomenclatural acts

The electronic version of this article in Portable Document Format (PDF) will represent a published work according to the International Commission on Zoological Nomenclature (ICZN), and hence the new name contained in the electronic version are effectively published under that Code from the electronic edition alone. This published work and the nomenclatural acts it contains have been registered in ZooBank, the online registration system for the ICZN. The ZooBank LSIDs (Life Science Identifiers) can be resolved and the associated information viewed through any standard web browser by appending the LSID to the prefix http://zoobank.org/. The LSID for this publication is: [urn:lsid:zoobank.org:pub:1AAA331C-C60D-47C2-8378-EEF3C33F7684]. The online version of this work is archived and available from the following digital repositories: PeerJ, PubMed Central and CLOCKSS.

### DNA extraction and sequencing

Seven individuals collected at Santiago Island were used for molecular analyses using two different approaches. For two individuals, DNA was extracted using DNAeasy Tissue and Blood Kit (Quiagen Inc., Valencia, CA, USA) following the manufacturer’s instructions. All extractions were visualized with a 1% agarose gel and quantified using an AccuClear UltraHigh Sensitivity dsDNA quantification kit (Biotium, Inc.) and SpectraMax M2 microplate reader. A restriction site associated DNA sequencing protocol (ezRAD; see Toonen et al., 2013; Knapp et al., 2016) was used for sequencing using the GATC cut site restriction enzyme *DpnII* in 50 µl reactions following manufacturer’s instructions, and then by 3 h incubation at 37 °C and 20 min at 65 °C. Samples were cleaned with Ampure XP beads in 1:1.8 ratio of DNA:beads and libraries were generated using KAPA HyperPrep library preparation kit (Roche) including the size-selection (350–700 bp) from Knapp et al. (2016) and PCR steps based on manufacturers recommendations. All libraries were sequenced as 300 bp single-end reads on the Illumina MiSeq platform at the Genetics Core Facility of the Hawai’i Institute of Marine Biology. All sequences were trimmed for quality and adaptors and assembled with the usage of a reference sequence (AQ2 *Tubastraea coccinea* HG965344, HG965278 and HG965410) to recover two mitochondrial and one nuclear markers using the default settings on Geneious 11.1.5 (https://www.geneious.com) (Kearse et al., 2012). The three target regions were (1) cytochrome c oxidades subunit I (COI), (2) an intragenic region between COI and trnM, trnM and a portion of the large ribosomal subunit (hereinafter called IGR), and (3) ITS1, 5.8S, ITS2 2 and a portion of 18S and 28S (herein called rDNA).

The remaining five individuals from Santiago Island had their DNA extracted using ReliaPrep™ gDNA Tissue Miniprep System - Promega and the three target genes were amplified by Polymerase Chain Reaction (PCR) in 10 µl solution containing 5 µl of TopTaq Master Mix (1.5 U taq polymerase, 3 mM MgCl_2_ and 400 µM of each dNTP), 4.1 µl of distilled water, 0.2 µM for both primers, and ∼20 ng of DNA. COI (∼600 bp) was amplified using the primers LCO1490 (5′-GGTCAACAAATCATAAAGATATTGG-3′) and HCO2198 (5′-TAAACTTCAGGGTGACCAAAAAATCA-3′) (Folmer et al., 1994). IGR (∼900 bp) was amplified using the primers CS 18F (5′-GGACACAAGAGCATATTTTACTG-3′) and CS 18R (5′-CTACTTACGGAATCTCGTTTGA -3′) (Lin et al., 2011). ITS region (∼980 bp) was amplified by using the primers 1S (5′-GGTACCCTTTGTACACACCGCCCGTCGCT-3′) and 2SS (5′-GCTITGGGCTGCAGTCCCAAGCAACCCGACTC-3′) (Chen, Willis & Miller, 1996).

The four samples from São Vicente Island had their DNA extracted following the procedures outlined in López et al. (2015) and two target genes (COI and rDNA) were amplified by Polymerase Chain Reaction (PCR) using AmpONE Taq DNA polymerase (GeneAll Biotechnology, South Korea) and following the manufacturer’s instructions. COI was amplified using the primers Lc2COI (5′-CGTTATTTTAGTATTTGGGATTGG-3′) (Hellberg, 2006) and HCO2198 (5′-TAA ACT TCA GGG TGA CCA AAA AAT CA-3′) (Folmer et al., 1994). rDNA was amplified using the primers A18S (5′-GATCGAACGGTTTAGTGAGG-3′) (Takabayashi et al., 1998) and ITS4 (5′-TCCTCCGCTTATTGATATGC-3′) (White et al., 1990).

Cycling conditions of all amplifications are described in Table 1. PCR products were purified with ExoSAP-IT according to the manufacturer’s instructions. Samples were sequenced on ABI 3500 Series Genetic Analyzer at a private company in Brazil (ACTGene Análises Moleculares) and at the Genomic Service (SEGAI) of the University of La Laguna. All sequences were deposited at GenBank (File S2). DNA sequences were edited using MEGA7 (Kumar, Stecher & Tamura, 2016) and Geneious 11.1.5 (Kearse et al., 2012).

**Table 1:** Cycling conditions used to amplify the three target regions (COI, cytochrome c oxidades subunit I; rDNA—ITS1, 5.8S, ITS2 2 and a portion of 18S and 28S and IGR—an intragenic region between COI and trnM, trnM and a portion of the large ribosomal subunit) from two localities, Tarrafal and Mindelo.

	COI		rDNA		IGR
	Tarrafal	Mindelo	Tarrafal	Mindelo	Tarrafal
Cycling conditions	3 min-95 °C	2 min-94 °C	3 min-95 °C	2 min-94 °C	3 min-95 °C
	**35x**	**40x**	**4x**	**40x**	**5x**
	**30 s-94 °C**	**10 s-94 °C**	**30 s-94 °C**	**10 s-94 °C**	**30 s-94 °C**
	**30 s-48 °C**	**20 s-60 °C**	**45 s-65 °C**	**20 s-54 °C**	**60 s-65 °C**
	**60 s-72 °C**	**30 s-72 °C**	**75 s-72 °C**	**30 s-72 °C**	**120 s-72 °C**
	10 min-72 °C	10 min-72 °C	**25x**	10 min-72 °C	**35x**
			**30 s-94 °C**		**30 s-94 °C**
			**45 s-60 °C**		**60 s-60 °C**
			**75 s-72 °C**		**120 s-72 °C**
			10 min-72 °C		5 min-72 °C

### Phylogenetic analyses

Additional sequences of 67 dendrophylliids and one poritid species (used as outgroup) were downloaded from GenBank for phylogenetic analyses (File S2) and two phylogenetic analyses were performed, the first including only samples from Santiago Island (*n* = 7), amplified by all three target genes (COI, IGR and rDNA), and the second including samples from both Santiago and São Vicente Islands (*n* = 11), amplified for two target genes (COI and rDNA). For the first phylogeny, sequences were aligned using MUSCLE implemented in Geneious 11.1.5 (Kearse et al., 2012) and concatenated in a final alignment of 1,845 bp in length. Maximum Likelihood (ML) and Bayesian inference (BI) reconstructions were performed using PhyML (Guindon et al., 2010) and MrBayes 3.2.6 (Ronquist & Huelsenbeck, 2003) available in Geneious. For the ML analyses the evolutionary model HKI+I was used, as suggested by jModelTest (Darriba et al., 2015) for the concatenated sequences with 100 bootstrap replicates. For the BI, specific evolutionary models were used for each locus as suggested by PartitionFinder 2 (Lanfear et al., 2012): HKY+I+G for COI and IGR; and TRNEF+I+G for the rDNA. Bayesian analyses were run for 1.1 million generations with sampling every 200 generations and a burn-in of 1,100,000. Methods applied for the second phylogenetic analyses are describe at File S3.

## Results

### Systematics

**Table:** 

Class Anthozoa Ehrenberg, 1834
Subclass Hexacorallia Haeckel, 1896
Order Scleractinia Bourne, 1900
Family Dendrophylliidae Gray, 1847
*Atlantia* gen. nov. López & Capel

**Type species**. *Atlantia caboverdiana* (Ocaña & Brito, 2015), by monotypy, here designated.

**Diagnosis**. Colonies bushy, phaceloid to dendroid, all achieved by extratentacular budding (frequently from theca of a parent corallite at an acute angle). No epitheca. Septa normally arranged and granular. Columella poorly to moderately developed.

**Remarks**. By having new corallites budding from the common basal coenosteum of the colony or from the edge zone of corallites, in gross morphology, *Atlantia* gen. nov. is morphologically more similar to the following dendrophylliid genera: *Cladopsammia* Lacaze-Duthiers, 1897; *Astroides* Quoy & Gaimard, 1827; *Enallopsammia* Sismonda, 1871; *Tubastraea* Lesson, 1829; and *Dendrophyllia* de Blainville, 1830. The new genus differs from those and other dendrophylliid genera by being always attached, having normally arranged septa (Portualès Plan absent), a poorly developed columella and displaying an uniform corallum porosity. Phylogenetic reconstructions recovered *A. caboverdiana* as an isolated clade, supporting the description of a new genus to better accommodate the species.

**Distribution**. Cape Verde archipelago, eastern Atlantic, 1–19 m depth (Ocaña et al., 2015, present results).

**Etymology**. Named in allusion to the Atlantic Ocean.

*Atlantia caboverdiana* (Ocaña & Brito, 2015), new combination

Figs. 1–3

**Figure 1:**
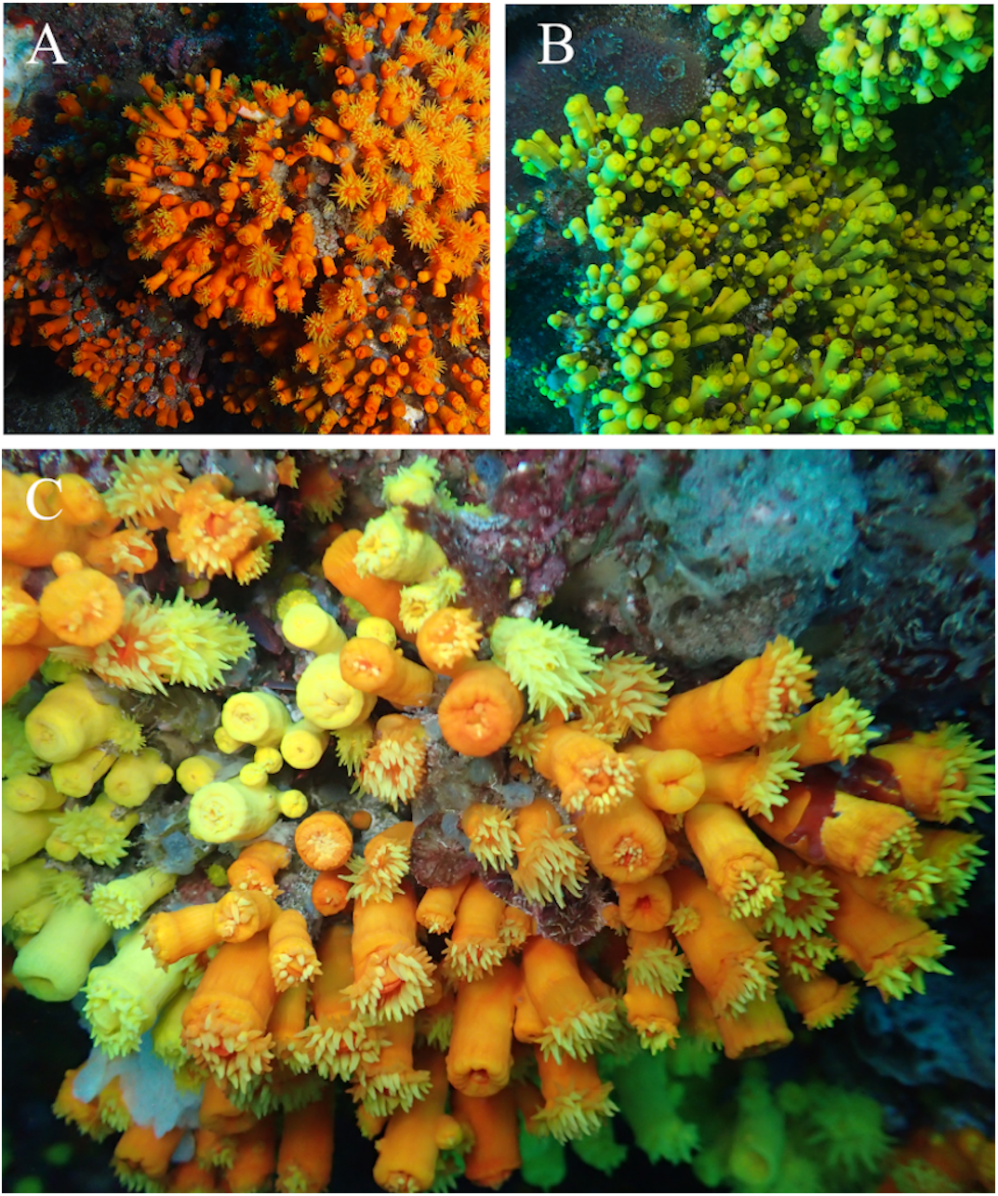
*In situ* images of *Atlantia caboverdiana* at Cape Verde. *In situ* images of *Atlantia caboverdiana* at Cape Verde. (A) orange color morph; (B) yellow color morph; and (C) Both color morphs growing together. Images courtesy from Oscar Ocaña Vicente.

**Figure 2:**
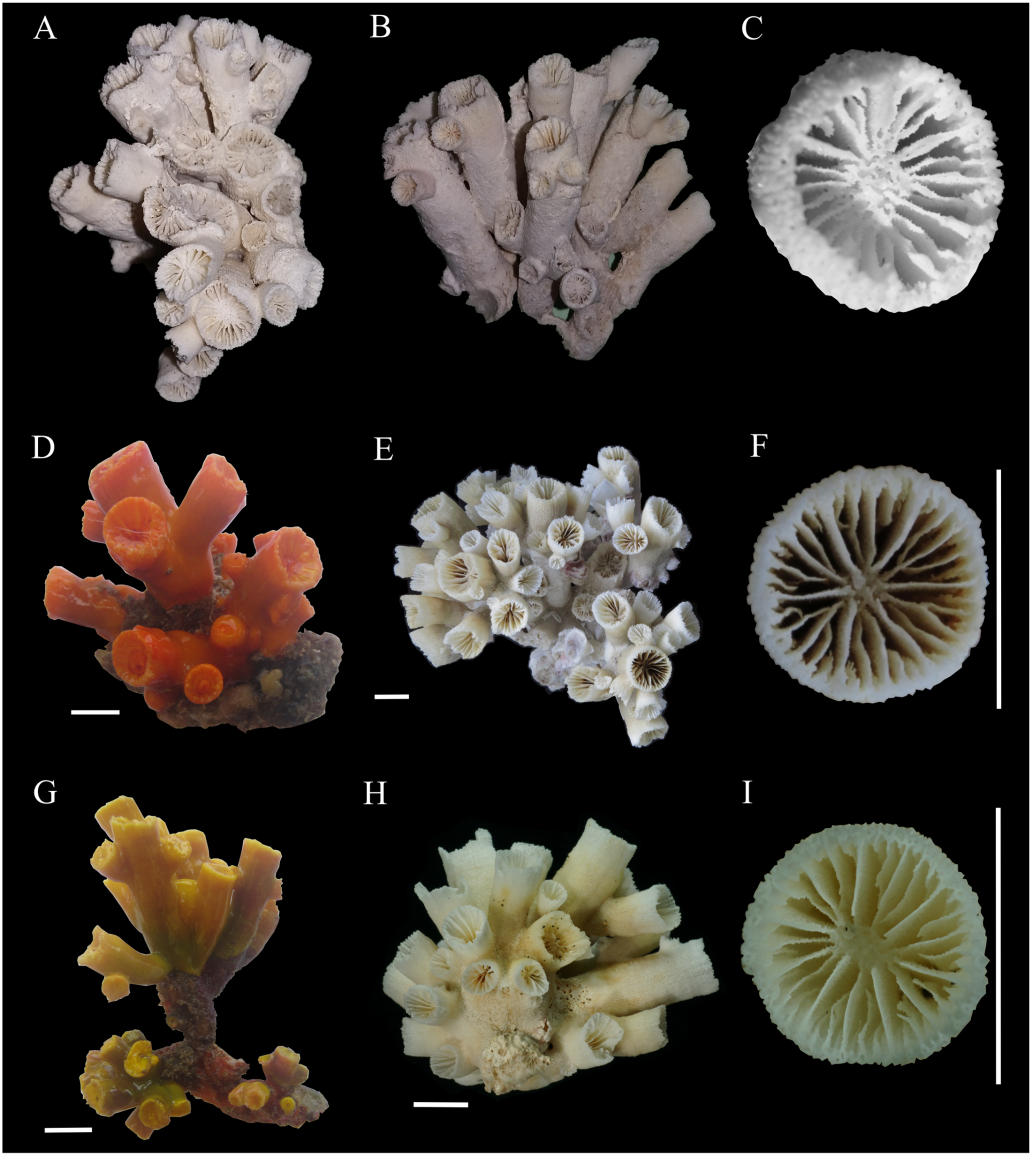
Colonies and corallites of *Atlantia caboverdiana*. *Atlantia caboverdiana* view of colonies and corallites. (A–C) holotype of *Atlantia caboverdiana* (MMC-26), deposited at *Museo del mar de Ceuta* (MMC) (Spain); (D) orange color morph CVL-1; (E–F) orange color morph CVL-3; (G) yellow color morph CVA-10; and (H–I) yellow color morph CVA-11. Scale bars: 1 cm. Holotype images courtesy from Oscar Ocaña Vicente.

**Figure 3:**
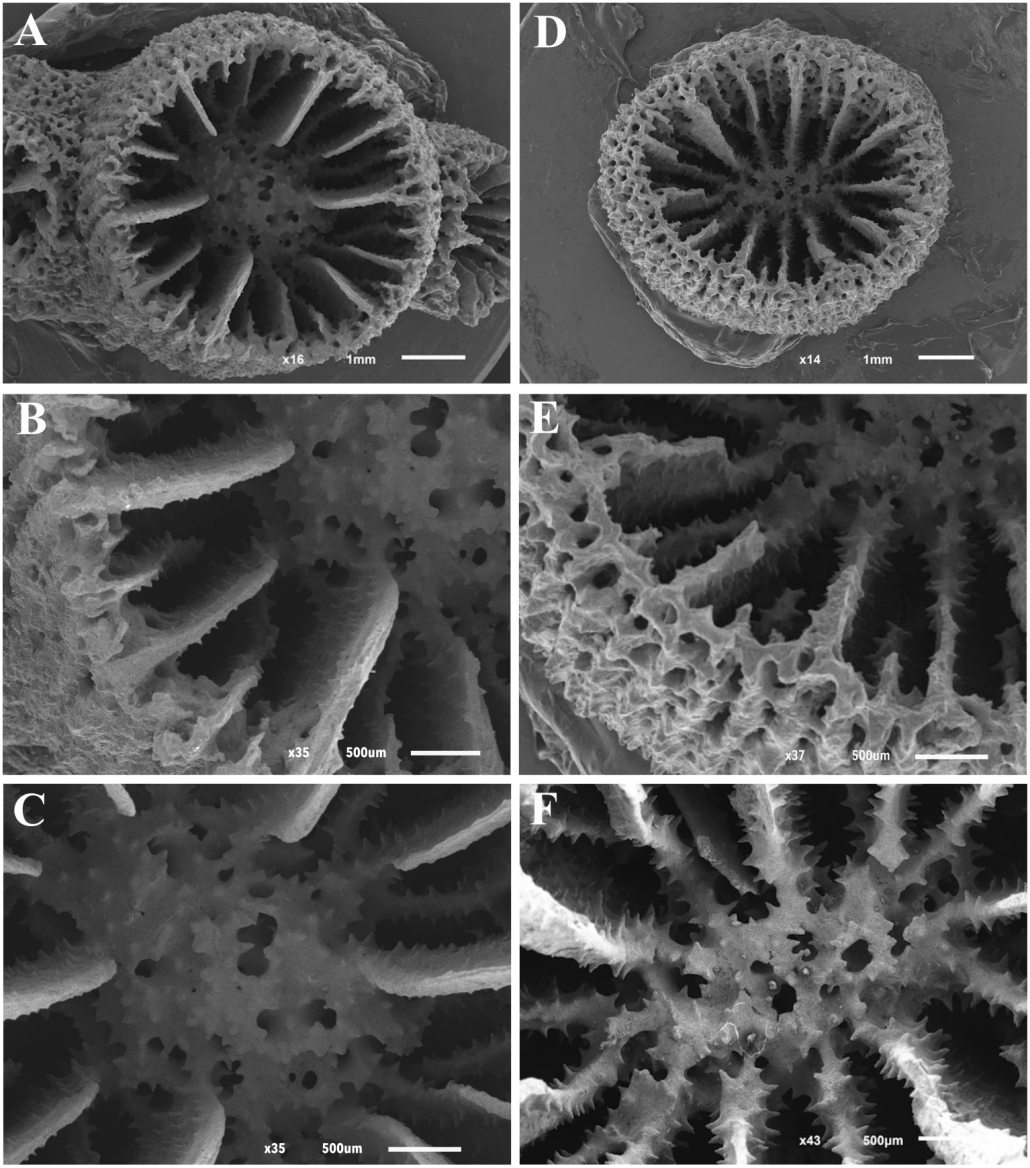
Scanning electron microscope (SEM) images of *Atlantia caboverdiana*. Scanning electron microscope (SEM) images of *Atlantia caboverdiana* from Santiago Island, Cape Verde, showing details of septa and columella. (A–C) specimen CVL-13 and (D–F) specimen CVA-10.

*Enallopsammia micranthus*—Chevalier (1966): 1387–1390.

*Tubastraea* sp.—(Laborel, 1974): 434–435.

*Tubastrea caboverdiana* Ocaña & Brito, 2015: 48–52.

**Type material**. MMC-26 (Holotype) (Ocaña et al., 2015).

**Type locality**. Santiago Island, Cape Verde, 10 m depth.

**Material examined**. Tarrafal, Santiago Island, Cape Verde, 21 colonies of which 11 displayed tissue yellow pigmented and 10 displayed tissue orange pigmented.

**Taxonomic history**. This species was first identified as *Enallopsammia micranthus* by Chevalier (1966) and later moved to *Tubastraea* by Laborel (1974). A species level identification was given only recently by Ocaña et al. (2015), who described it as “*Tubastrea caboverdiana”*. The species is herein re-described as *Atlantia caboverdiana*.

**Distribution**. Currently known only from the Cape Verde archipelago but, based on descriptions from Laborel (1974), *A. caboverdiana* possibly occurs also in the Gulf of Guinea.

**Description**. Corallum phaceloid to dendroid forming bushy colonies. Budding extratentacular from corallum base and also from theca of a parent corallite. The largest colony examined bears 89 corallites. Corallite cylindrical; calice circular to slightly elliptical ranging between 3 and 11 mm in largest calicular diamenter. Most examined colonies bear a few main corallites projecting up to 56 mm above base, from which new buds arise. Calicular edge slightly thinner than remaining theca. Theca porous especially near calicular edge. Costae granular, separated by deep narrow ridges. Coenosarc orange or yellow. Tentacles always yellow in the yellow morph but orange and yellow on the orange morph. Corallum white.

Septa hexamerally arranged in four nonexsert cycles according to the formula: S1>S2>S3>S4. All septa thin. S1 extend about 2/3 distance to columella with entire and vertical axial edge. S2 slightly smaller than S1. S1-2 fuse to columella deep in fossa. S3 about $ \frac{1}{2} $ width of S2. In each system, a pair of S3 fuses to S2 near columella. S3 axial edge laciniate in small corallites but entire in larger corallites. S4 rudimentary, entire or having slightly laciniate axial edge. Septal faces covered with pointed granules. Fossa deep containing a poorly or sometimes moderately developed spongy columella.

**Remarks.** *Atlantia caboverdiana* differs from *Tubastraea* representatives by having a phaceloid to dendroid corallum forming a bushy colony with new corallites budding from the theca of a parent corallite in an acute angle. Septa width also differentiates *Atlantia* from *Tubastraea* species; septa are wider and project further into the calice in *Atlantia*. Two morphologically indistinguishable *Atlantia caboverdiana* color morphs are found in Cape Verde, a yellow and an orange one. According to Laborel (1974), the specimens from Cape Verde resemble a yellow morph found in the Gulf of Guinea. SEM images show no clear difference between the two color morphs, with all septa covered by sharp spines and columella covered by round to sharp granules (Fig. 3). Our phylogeny reconstruction recovered *A. caboverdiana* as more closely related to *Dendrophyllia cornigera* (Lamarck, 1816) and *Leptopsammia pruvoti* Lacaze-Duthiers, 1897 and distantly related to *Tubastraea*.

### Phylogenetic analyses

**Figure 4:**
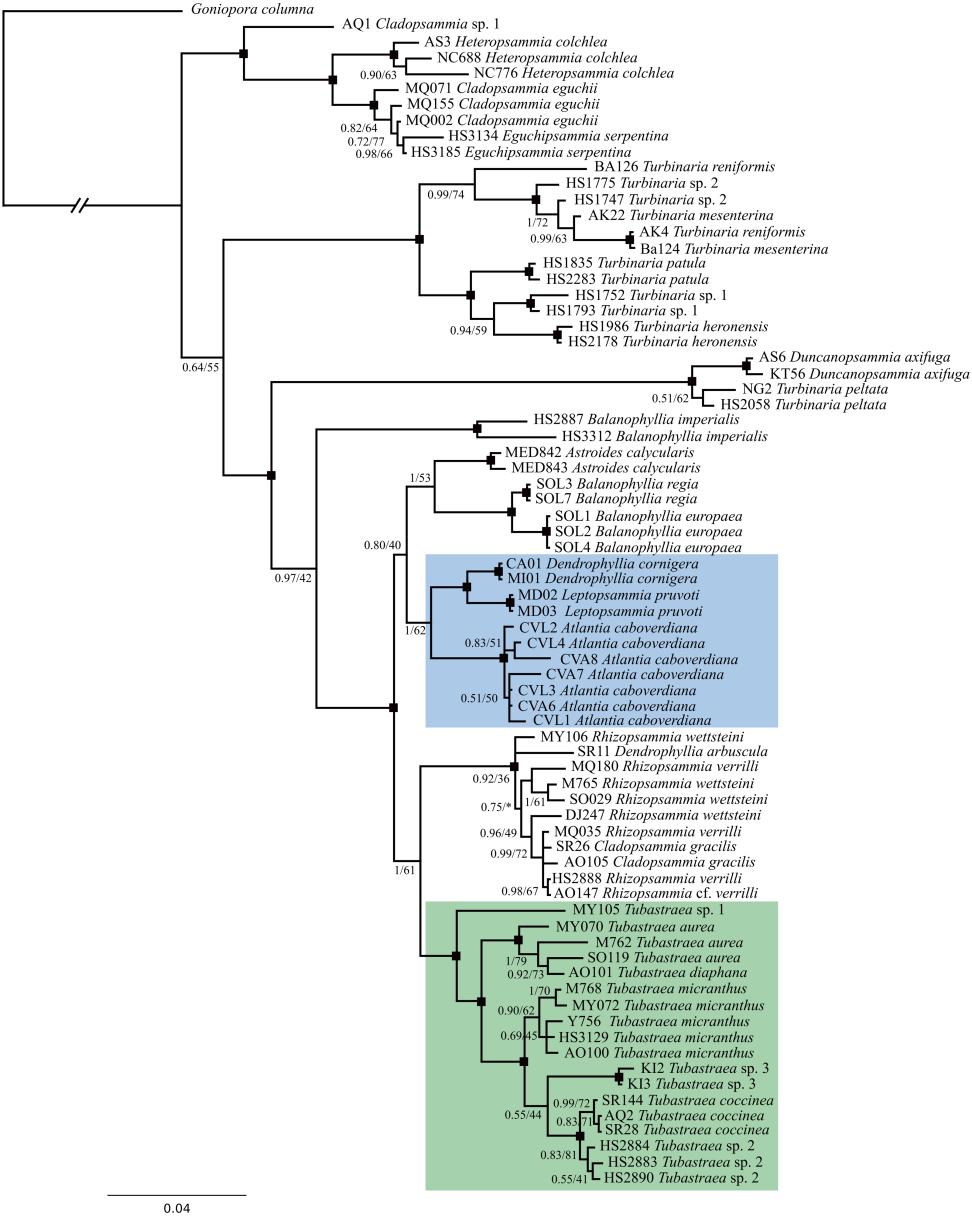
A phylogeny reconstruction of Dendrophylliidae. Phylogenetic analyses based on Bayesian inference of the concatenated genes COI, IGR and rDNA from 75 dendrophylliid corals and *Goniopora columna* as external group. Black dots indicate branches with Posterior probability ≥95 and bootstrap support value ≥85. An asterisk (*) indicates a branch recovered on a different position by Maximum likelihood analyses.

Phylogenetic analyses were performed based on two mitochondrial (COI and IGR) and one nuclear (rDNA) marker, having 557, 448 and 840 bp, respectively. Sequences were concatenated in a final alignment of 1,845 bp for a total of 31 species represented by 75 specimens. Within these partial sequences the rDNA displayed the highest phylogenetic signal and both BI and ML recovered nearly identical topologies. The same topology was also recovered when only COI and rDNA were used for phylogenetic reconstructions (File S4), corroborating that *Atlantia caboverdiana* does not belong to the genus *Tubastraea*. In all evolutionary reconstructions *Atlantia caboverdiana* was recovered more closely related to a clade containing *Dendrophyllia cornigera* and *Leptopsammia pruvoti* (Fig. 4).

## Discussion

Although morphological and molecular data corroborates the monophyly of Dendrophylliidae (Cairns, 2001; Arrigoni et al., 2014), several genera within the family appear to be poly- or paraphyletic (Kitahara et al., 2010; Arrigoni et al., 2014). Dendrophylliidae is the third most diverse family within the order Scleractinia and has an intricate and challenging taxonomy. Morphological plasticity and intraspecific variability plus evolutionary convergence and homoplasy are some of the factors that frequently challenge traditional scleractinian taxonomy, especially for shallow-water species (Kitahara et al., 2010). Hence, based on both morphological characters and molecular data, we erect *Atlantia* as a new genus in the family Dendrophylliidae, which is currently known to occur only in the sub-tropical East Atlantic Ocean.

Despite sharing morphological similarities with *Cladopsammia*, *Astroides*, *Enallopsammia* and *Dendrophyllia*, *Atlantia caboverdiana* does not fit within any of these or other Dendrophylliidae genera. According to the diagnosis, *Cladopsammia* form “*small bushy colonies formed by extratentacular budding from common basal coenosteum and occasionally from edge zone of larger corallites…Pourtalès plan well developed*” (Cairns, 2001). However, although budding from the thecal edge of larger corallites is frequent, the Pourtalès Plan is absent in *A. caboverdiana*. On the recovered phylogeny reconstruction, *Cladopsammia* is polyphyletic and none of the three species included appears to be closely related to *A. caboverdiana*. *Astroides* is a monospecific genus with variable morphology (cerioid, plocoid and phaceloid), characterized by having a massive columella, a shallow fossa and septa with dentate axial edges (Cairns, 2001), none of which were observed in *A. caboverdiana*. Furthermore, no close relationship with this genus was recovered by molecular data. *Dendrophyllia*, another genus sharing some morphological similarities to *Atlantia* (by having a bushy colony), is also polyphyletic (Arrigoni et al., 2014) and morphologically divided into three groups according to the colony growth form: monopodial, sympodial, and bushy (Cairns, 2009). All *Dendrophyllia* have septa arranged according to the Pourtalès Plan, which as stated above, is absent in *A. caboverdiana.*

Phylogenetic analyses comprising 11 of the 22 recognized genera within the family recovered *A. caboverdiana* as more closely related to *Dendrophyllia cornigera* and *Leptopsammia pruvoti*, both found in the Northeastern Atlantic (Zibrowius, 1980)*. Dendrophyllia cornigera* has a ramose growth form, somewhat similar to *A. caboverdiana*, but differs by having septa arranged in a Pourtalès Plan and a deeper distributional range (98–600 m depth) (Cairns, 2009). On the other hand, *Leptopsammia pruvoti* has normally arranged septa (not Portualès Plan) and primary and secondary septal cycles (S1 and S2) with smooth axial edges (Cairns, 2001); however, *Leptopsammia* refers to solitary species. Although lacking half of the extant genus diversity, our phylogeny reconstruction includes representatives of almost all colonial genera, except for *Dichopsammia* Song, 1994 and *Enallopsammia* Sismonda, 1871. *Dichopsammia* is a monospecific genus reported only in the North Pacific, with colonies formed exclusively by intratentacular budding. *Enallopsammia*, on the other hand, shows mostly extratentacular budding but all of its extant representatives have arborescent growth forms.

Therefore, both morphological and molecular similarities distinguish *A. caboverdiana* from all known dendrophylliid genera, justifying its placement into a new genus and supporting *Tubastraea* as being native to the Indo-Pacific and introduced into the Atlantic Ocean. Currently, the distribution of the new genus is restricted to the Archipelago of Cape Verde, although *Atlantia* might also occur in the Gulf of Guinea based on descriptions by Laborel (1974).

## Conclusions

Azooxanthellate corals remains understudied compared to their symbiotic counterparts (Kitahara et al., 2016) and the status of the genus *Tubastraea* in the EA has remained under discussion for several decades (Laborel, 1974; Creed et al., 2017). The transfer of *T. caboverdiana* to the newly established genus *Altantia* indicates that *Tubastraea* is indeed non-native in the Atlantic Ocean. Furthermore, the descriptions of *Tubastraea* spp. in Laborel (1974) would suggest that: (1) *Atlantia caboverdiana* probably occurs in its yellow form on the Gulf of Guinea; and (2) two more distinctive varieties, orange or yellow in color, may also be present on the continental African coast [supported by P. Wirtz, personal communication (Sierra Leone) and observations on oil platforms in Gabon (Friedlander et al., 2014)], probably pertaining to the genus *Tubastraea*. These observations support those by Ocaña et al. (2015) regarding the need for a re-evaluation of the eastern Atlantic shallow-water dendrophylliids.

##  Supplemental Information

10.7717/peerj.8633/supp-1 File S1 Specimens used for morphological comparison Details of specimens of *Atlantia caboverdiana* (Anthozoa, Dedrophylliidae) used for morphological comparison. All material is deposited at the Museu Nacional do Rio de Janeiro.

10.7717/peerj.8633/supp-2 File S2 List of species included in phylogenetic analyses List of specimens of Dendrophylliidae and Poritidae included in phylogenetic analyses with corresponding identification, locality and accession numbers. An asterisk (*) indicates new sequences obtained by the present study. Remaining sequences (except for *Goniopora columna*) are from Arrigoni et al. (2014).

10.7717/peerj.8633/supp-3 File S3 Methods applied for evolutionary reconstructions including two target regions (COI and rDNA) Description of the methods applied for evolutionary reconstructions including two target regions (COI and rDNA).

10.7717/peerj.8633/supp-4 File S4 Phylogenetic analyses of the concatenated regions COI and rDNA Phylogenetic analyses based on Bayesian inference (BI) and Maximum Likelihhod (ML) of the concatenated regions COI and rDNA from 74 Dendrophylliidae corals and *Goniopora columna* as external group. Values at branches represent posterior probabilities and bootstrap support for BI and ML analyses, respectively.

## Additional Information and Declarations

### Field Study Permissions

The following information was supplied relating to field study approvals (i.e., approving body and any reference numbers): This study was carried out under authorization No. 014/2015 from the Direcção Nacional do Ambiente, Cabo Verde. Samples were sent to Brazil under the CITES export permit 14/15/CITES/DNA and import permit 15BR017494/DF.

### Data Availability

The following information was supplied regarding data availability: The COI sequences are available at GenBank: MN414205 to MN414211 and MN384731 to MN384734. The IGR sequences are available at GenBank: MN414213 to MN414219. The rDNA sequences are available at GenBank: MN412646 to MN412652 and MN306195 to MN306198. All dry specimens from Santiago Island are deposited at the Museu Nacional do Rio de Janeiro (MNRJ 9108-9111; 9113-9115) (File S1). Pictures of the holotype of T. caboverdiana, available at the Museo del mar de Ceuta (MMC) (Spain) (MMC-26), were also used.

### New Species Registration

The following information was supplied regarding the registration of a newly described species: Publication LSID: urn:lsid:zoobank.org:pub:1AAA331C-C60D-47C2-8378-EEF3C33F7684 Atlantia LSID: urn:lsid:zoobank.org:act:66791E04-685E-4ED6-9780-B443C6A79147
