# Supplementary material for: Atlantia, a new genus of Dendrophylliidae (Cnidaria, Anthozoa, Scleractinia) from the eastern Atlantic
Source: PeerJ. 2020 Mar 16;8:e8633. doi: 10.7717/peerj.8633 (PMC7081789; doi:10.7717/peerj.8633)
Supplement: File S1 — Details of specimens of Atlantia caboverdiana (Anthozoa, Dedrophylliidae) used for morphological comparison. All material is deposited at the Museu Nacional do Rio de Janeiro. [file peerj-08-8633-s001.docx]

**Supplementary File 1**

Details of specimens of *Atlantia caboverdiana* (Anthozoa, Dedrophylliidae) used for morphological comparison. All material is deposited at the Museu Nacional do Rio de Janeiro.

| **Specie** | **Sample location** | **Depth** | **Color mprph** | **Museum number** | **ID sample for DNA** |
| --- | --- | --- | --- | --- | --- |
| *Atlantia caboverdiana* | Tarrafal, Santiago Island | 6-10 m | Orange | MNRJ 9108 | CVL1 |
| *Atlantia caboverdiana* | Tarrafal, Santiago Island | 6-10 m | Orange | MNRJ 9109 | CVL2 |
| *Atlantia caboverdiana* | Tarrafal, Santiago Island | 6-10 m | Orange | MNRJ 9110 | CVL3 |
| *Atlantia caboverdiana* | Tarrafal, Santiago Island | 6-10 m | Orange | MNRJ 9111 | CVL4 |
| *Atlantia caboverdiana* | Tarrafal, Santiago Island | 6-10 m | Yellow | MNRJ 9113 | CVA5 |
| *Atlantia caboverdiana* | Tarrafal, Santiago Island | 6-10 m | Yellow | MNRJ 9114 | CVA6 |
| *Atlantia caboverdiana* | Tarrafal, Santiago Island | 6-10 m | Yellow | MNRJ 9115 | CVA7 |
