# Supplementary material for: Atlantia, a new genus of Dendrophylliidae (Cnidaria, Anthozoa, Scleractinia) from the eastern Atlantic
Source: PeerJ. 2020 Mar 16;8:e8633. doi: 10.7717/peerj.8633 (PMC7081789; doi:10.7717/peerj.8633)
Supplement: File S2 — List of specimens of Dendrophylliidae and Poritidae included in phylogenetic analyses with corresponding identification, locality and accession numbers. An asterisk (*) indicates new sequences obtained by the present study. Remaining sequences (except for Goniopora columna) are from Arrigoni et al. (2014). [file peerj-08-8633-s002.docx]

**Supplementary File 2**

List of specimens of Dendrophylliidae and Poritidae included in phylogenetic analyses with corresponding identification, locality and accession numbers. * indicates new sequences obtained by the present study. Remaining sequences (except for *Goniopora columna*) are from Arrigoni et al. (2014).

| **Species** | **Identification** | **Locality** | **Accession numbers** | | |
| --- | --- | --- | --- | --- | --- |
|  |  |  | **COI** | **IGR** | **rDNA** |
| **Dendrophylliidae** |  |  |  |  |  |
| *Atlantia caboverdiana* * | CVL-1 | Cape Verde | MN414205 | MN414213 | MN412646 |
| *Atlantia caboverdiana* * | CVL-2 | Cape Verde | MN414206 | MN414214 | MN412647 |
| *Atlantia caboverdiana* * | CVL-3 | Cape Verde | MN414207 | MN414215 | MN412648 |
| *Atlantia caboverdiana* * | CVL-4 | Cape Verde | MN414208 | MN414216 | MN412649 |
| *Atlantia caboverdiana* * | CVA-6 | Cape Verde | MN414209 | MN414217 | MN412650 |
| *Atlantia caboverdiana* * | CVA-7 | Cape Verde | MN414210 | MN414218 | MN412651 |
| *Atlantia caboverdiana* * | CVA-8 | Cape Verde | MN414211 | MN414219 | MN412652 |
| *Atlantia caboverdiana* * | CV304 | Cape Verde | MN384731 | - | MN306195 |
| *Atlantia caboverdiana* * | CV305 | Cape Verde | MN384732 | - | MN306196 |
| *Atlantia caboverdiana* * | CV315 | Cape Verde | MN384733 | - | MN306197 |
| *Atlantia caboverdiana* * | CV316 | Cape Verde | MN384734 | - | MN306198 |
| *Astroides calycularis* | MED842 | Mediterranean Sea | HG965307 | HG965239 | HG965371 |
| *Astroides calycularis* | MED843 | Mediterranean Sea | HG965308 | HG965240 | HG965372 |
| *Balanophyllia* (*Balanophyllia*) *europaea* | SOL1 | Mediterranean Sea | HG965309 | HG965241 | HG965373 |
| *Balanophyllia* (*Balanophyllia*) *europaea* | SOL2 | Mediterranean Sea | HG965310 | HG965242 | HG965374 |
| *Balanophyllia* (*Balanophyllia*) *europaea* | SOL4 | Mediterranean Sea | HG965311 | HG965243 | HG965375 |
| *Balanophyllia* (*Eupsammia*) *imperialis* | HS3312 | New Caledonia | HG965312 | HG965244 | HG965376 |
| *Balanophyllia* (*Eupsammia*) *imperialis* | HS2887 | New Caledonia | HG965313 | HG965245 | HG965377 |
| *Balanophyllia* (*Balanophyllia*) *regia* | SOL3 | Mediterranean Sea | HG965314 | HG965246 | HG965378 |
| *Balanophyllia* (*Balanophyllia*) *regia* | SOL7 | Mediterranean Sea | HG965315 | HG965247 | HG965379 |
| *Cladopsammia* sp. 1 | AQ1 | Japan | HG965316 | HG965248 | HG965380 |
| *Cladopsammia eguchii* | MQ002 | Marquesas, French Polynesia | HG965317 | HG965249 | HG965381 |
| *Cladopsammia eguchii* | MQ071 | Marquesas, French Polynesia | HG965318 | HG965250 | HG965382 |
| *Cladopsammia eguchii* | MQ155 | Marquesas, French Polynesia | HG965319 | HG965251 | HG965383 |
| *Cladopsammia gracilis* | AO105 | Japan | - | HG965252 | HG965384 |
| *Cladopsammia gracilis* | SR26 | Japan | HG965320 | HG965253 | HG965385 |
| *Dendrophyllia arbuscula* | SR11 | Japan | HG965321 | HG965254 | HG965386 |
| *Dendrophyllia cornigera* | CA01 | Mediterranean Sea | HG965322 | HG965255 | HG965387 |
| *Dendrophyllia cornigera* | MI01 | Mediterranean Sea | HG965323 | HG965256 | HG965388 |
| *Duncanopsammia axifuga* | AS6 | Australia | HG965325 | HG965258 | HG965390 |
| *Duncanopsammia axifuga* | KT56 | Australia | HG965326 | HG965259 | HG965391 |
| *Eguchipsammia* *serpentina* | HS3134 | New Caledonia | HG965327 | HG965260 | HG965392 |
| *Eguchipsammia* *serpentina* | HS3185 | New Caledonia | HG965328 | HG965261 | HG965393 |
| *Heteropsammia cochlea* | AS3 | Australia | HG965329 | HG965262 | HG965394 |
| *Heteropsammia cochlea* | NC688 | New Caledonia | HG965330 | HG965263 | HG965395 |
| *Heteropsammia cochlea* | NC776 | New Caledonia | HG965331 | HG965264 | HG965396 |
| *Leptopsammia pruvoti* | MD02 | Mediterranean Sea | HG965332 | HG965265 | HG965397 |
| *Leptopsammia pruvoti* | MD03 | Mediterranean Sea | HG965333 | HG965266 | HG965398 |
| *Rhizopsammia* cf *verrilli* | AO147 | Japan | - | HG965267 | HG965399 |
| *Rhizopsammia verrilli* | MQ035 | Marquesas, French Polynesia | HG965334 | HG965268 | HG965400 |
| *Rhizopsammia verrilli* | MQ180 | Marquesas, French Polynesia | HG965335 | HG965269 | HG965401 |
| *Rhizopsammia verrilli* | HS2888 | New Caledonia | HG965336 | HG965270 | HG965402 |
| *Rhizopsammia wettsteini* | DJ247 | Djibouti | HG965337 | HG965271 | HG965403 |
| *Rhizopsammia wettsteini* | M765 | Maldives | HG965338 | HG965272 | HG965404 |
| *Rhizopsammia wettsteini* | MY106 | Mayotte Island | HG965339 | HG965273 | HG965405 |
| *Rhizopsammia wettsteini* | SO029 | Socotra Island, Yemen | HG965340 | HG965274 | HG965406 |
| *Tubastraea cf aurea* | M762 | Maldives | HG965341 | HG965275 | HG965407 |
| *Tubastraea cf aurea* | MY070 | Mayotte Island | HG965342 | HG965276 | HG965408 |
| *Tubastraea cf aurea* | SO119 | Socotra Island, Yemen | HG965343 | HG965277 | HG965409 |
| *Tubastraea coccinea* | AQ2 | Japan | HG965344 | HG965278 | HG965410 |
| *Tubastraea coccinea* | SR144 | Japan | HG965345 | HG965279 | HG965411 |
| *Tubastraea coccinea* | SR28 | Japan | HG965346 | HG965280 | HG965412 |
| *Tubastraea diaphana* | AO101 | Japan | - | HG965281 | HG965413 |
| *Tubastraea micranthus* | AO100 | Japan | - | HG965282 | HG965414 |
| *Tubastraea micranthus* | HS3129 | New Caledonia | HG965347 | HG965283 | HG965415 |
| *Tubastraea micranthus* | M768 | Maldives | HG965348 | HG965284 | HG965416 |
| *Tubastraea micranthus* | MY072 | Mayotte Island | HG965349 | HG965285 | HG965417 |
| *Tubastraea micranthus* | Y756 | Yemen | HG965350 | HG965286 | HG965418 |
| *Tubastraea* sp. 1 | MY105 | Mayotte Island | HG965351 | HG965287 | HG965419 |
| *Tubastraea* sp. 2 | HS2883 | New Caledonia | HG965352 | HG965288 | HG965420 |
| *Tubastraea* sp. 2 | HS2884 | New Caledonia | HG965353 | HG965289 | HG965421 |
| *Tubastraea* sp. 2 | HS2890 | New Caledonia | HG965354 | HG965290 | HG965422 |
| *Tubastraea* sp. 3 | KI2 | Japan | HG965355 | HG965291 | HG965423 |
| *Tubastraea* sp. 3 | KI3 | Japan | HG965356 | HG965292 | HG965424 |
| *Turbinaria heronensis* | HS1986 | New Caledonia | HG965357 | HG965293 | HG965425 |
| *Turbinaria heronensis* | HS2178 | New Caledonia | HG965358 | HG965294 | HG965426 |
| *Turbinaria mesenterina* | AK22 | Japan | HG965359 | HG965295 | HG965427 |
| *Turbinaria mesenterina* | BA124 | Yemen | HG965360 | HG965296 | HG965428 |
| *Turbinaria patula* | HS2283 | New Caledonia | HG965361 | HG965297 | HG965429 |
| *Turbinaria patula* | HS1835 | New Caledonia | HG965362 | HG965298 | HG965430 |
| *Turbinaria peltata* | HS2058 | New Caledonia | HG965363 | HG965299 | HG965431 |
| *Turbinaria peltata* | NG2 | Japan | HG965364 | HG965300 | HG965432 |
| *Turbinaria reniformis* | AK4 | Japan | HG965365 | HG965301 | HG965433 |
| *Turbinaria reniformis* | BA126 | Yemen | HG965366 | HG965302 | HG965434 |
| *Turbinaria* sp. 1 | HS1752 | New Caledonia | HG965367 | HG965303 | HG965435 |
| *Turbinaria* sp. 1 | HS1793 | New Caledonia | HG965368 | HG965304 | HG965436 |
| *Turbinaria* sp. 2 | HS1747 | New Caledonia | HG965369 | HG965305 | HG965437 |
| *Turbinaria* sp. 2 | HS1775 | New Caledonia | HG965370 | HG965306 | HG965438 |
| **Poritidae** |  |  |  |  |  |
| *Goniopora columna* | Y698 | Yemen | JF825141 | JF825141 | AB906954 |
