# Supplementary material for: Atlantia, a new genus of Dendrophylliidae (Cnidaria, Anthozoa, Scleractinia) from the eastern Atlantic
Source: PeerJ. 2020 Mar 16;8:e8633. doi: 10.7717/peerj.8633 (PMC7081789; doi:10.7717/peerj.8633)
Supplement: File S3 — Description of the methods applied for evolutionary reconstructions including two target regions (COI and rDNA). [file peerj-08-8633-s003.docx]

**Supplementary File 3**

Methods applied for evolutionary reconstructions including two target regions (COI and rDNA).

**Phylogenetic analyses**

Sequence alignment was performed using CLUSTAL W (Thompson et al. 1994) as implemented in MEGA7. The best-fitting model of nucleotide substitution for each data set and codon position (COI) was selected in jModeltest (Darriba et al. 2015) according to the Bayesian Information Criterion (Schwarz 1978). Phylogenetic trees of *Tubastraea caboverdiana* were inferred from the concatenated COI and ITS-rDNA sequences by maximum likelihood (ML) and Bayesian inference (BI), using the previously determined models of nucleotide evolution (K80 for COI, and K80+I+G for ITS-rDNA). ML analyses were conducted using RAxML software (Stamakatis 2014), with the GTR+CAT approximation to accommodate heterogeneity rate among partitions and 1,000 replicates of bootstrap (BS). For BI we used MrBayes software (Huelsenbeck and Ronquist 2001). In this case, two independent runs were performed with default prior values, running 10^6^ generations with sampling frequency every 100 generations. All parameters were unlinked across partitions. Convergence of all parameters in the two independent runs was assessed using Tracer 1.5 software (Rambaut and Drummond 2007). After removing 25% of samples as a burn-in, the remaining trees were used to obtain a majority consensus tree and only those nodes with posterior probabilities (PP) higher than 0.95 were considered significant (Huelsenbeck et al. 2001). ML and BI phylogenetic trees were generated using CIPRES Science Gateway V. 3.3: Cyberinfrastructure for Phylogenetic Research (Miller et al. 2010). Finally, trees were visualized and edited with Figtree v1.4.3 (Rambaut 2016).
