# Supplementary figures and images for: Atlantia, a new genus of Dendrophylliidae (Cnidaria, Anthozoa, Scleractinia) from the eastern Atlantic
Source: PeerJ. 2020 Mar 16;8:e8633. doi: 10.7717/peerj.8633 (PMC7081789; doi:10.7717/peerj.8633)

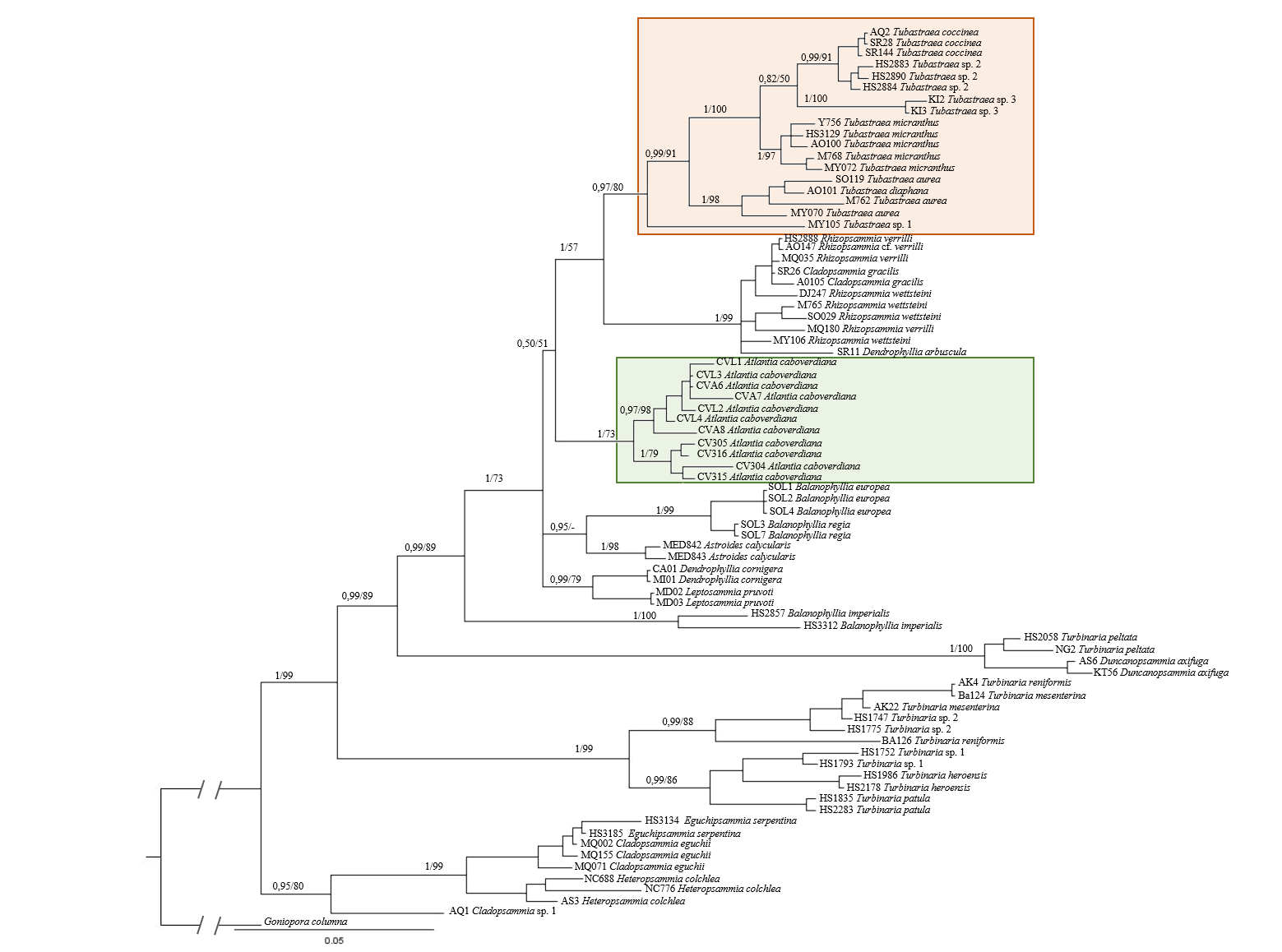

Supplement: File S4 — Phylogenetic analyses based on Bayesian inference (BI) and Maximum Likelihhod (ML) of the concatenated regions COI and rDNA from 74 Dendrophylliidae corals and Goniopora columna as external group. Values at branches represent posterior probabilities and bootstrap support for BI and ML analyses, respectively. [file peerj-08-8633-s004.png]
